# Post-Implant Global Longitudinal Strain as a Predictor of Pacing-Induced Cardiomyopathy in Patients with Preserved Ejection Fraction Undergoing Pacemaker Placement

**DOI:** 10.31083/RCM26173

**Published:** 2025-01-17

**Authors:** Sung Soo Kim, Hyung Wook Park, Hyung Ki Jeong

**Affiliations:** ^1^Department of Cardiovascular Medicine, Chosun University Medical School, 61469 Gwangju, Republic of Korea; ^2^Department of Cardiovascular Medicine, Chonnam National University Medical School, 61469 Gwangju, Republic of Korea; ^3^Department of Cardiovascular Medicine, Wonkwang University Medical School, 54536 Iksan, Republic of Korea

**Keywords:** echocardiography, heart failure, pacemaker, artificial

## Abstract

**Background:**

Right ventricular (RV) pacing exacerbates heart failure and increases cardiac mortality in patients with reduced ejection fraction (EF). However, its impact on left ventricular dysfunction in patients with preserved EF remains inconclusive. This study investigates the relationship between RV pacing, global longitudinal strain (GLS), and EF in patients with preserved EF.

**Methods:**

This prospective registry study included patients with preserved EF (≥50%) undergoing de novo permanent pacemaker (PPM) implantation for atrioventricular block at Chosun University Hospital, South Korea, from 2018 to 2022. Echocardiographic evaluations were performed pre-implant, post-implant, and at 12 months, with follow-up visits every 3–6 months. Composite outcomes included cardiac death, heart failure hospitalization, pacing-induced cardiomyopathy (PICM), and biventricular pacing (BVP) upgrade.

**Results:**

A total of 71 patients (28 males, mean age 73.1 years) were included. Following PPM implantation, significant declines in both EF and GLS were noted, especially in those with PICM. Over three years, 2 patients died, 6 were hospitalized, 7 developed PICM, and 3 underwent a BVP upgrade. Reduced post-implant GLS was an independent predictor of PICM (hazard ratios (HR) 1.715, 95% CI 1.174–2.504; *p* = 0.005). Receiver operating characteristic (ROC) analysis showed an area under curve (AUC) of 0.92 for GLS, with a GLS <–15.0 having 100% sensitivity and 80.9% specificity for predicting PICM.

**Conclusions:**

Post-implant GLS is a reliable predictor of PICM in patients with preserved EF. Regular GLS monitoring can guide timely interventions, including guideline-directed medical therapy or BVP upgrades, to prevent deterioration and improve outcomes.

## 1. Introduction

Right ventricular (RV) pacing has long been associated with an increased risk of left ventricular dysfunction, particularly in patients with a high pacing burden [1, 2]. Pacing-induced cardiomyopathy (PICM), characterized by a decline in left ventricular ejection fraction (LVEF), is a well-documented phenomenon [3]. Studies report an incidence of 10–20% for PICM after 2–4 years of RV pacing, accompanied by increased risks of atrial fibrillation (AF), hospitalization for heart failure (HHF), and cardiac mortality [2, 4, 5, 6, 7, 8, 9]. RV pacing can exacerbate heart failure and elevate cardiac mortality in patients with pre-existing low LVEF [1, 2, 5, 10].

Despite these known risks, early identification of patients with preserved LVEF at risk for PICM remains a clinical challenge. Recent research has highlighted the potential of global longitudinal strain (GLS) as a more sensitive marker for early left ventricular dysfunction, resulting in earlier detection compared to LVEF. GLS measures myocardial deformation and can detect functional impairment before a significant decline in LVEF becomes evident [11, 12, 13]. While previous studies have demonstrated the association between reduced GLS and PICM, most have focused on 3 dimensional GLS or broader patient populations, with little focus on the early changes in GLS following pacemaker implantation [11, 12, 13]. Furthermore, detailed exploration of GLS changes in patients with preserved EF undergoing pacemaker implantation is still needed.

This study seeks to address these gaps by exploring the relationship between early post-implantation GLS changes and the development of PICM in patients with preserved ejection fraction (EF). By investigating the early dynamics of GLS, we sought to offer insights that could guide earlier interventions and improve clinical outcomes for at-risk patients.

## 2. Method

### 2.1 Study Population

This prospective registry study examined patients receiving permanent pacemakers at Chosun University Hospital in Gwangju, South Korea, between July 2018 and June 2022. Eligible participants were individuals aged 18 years or older who underwent dual-chamber pacemaker implantation due to atrioventricular block. Inclusion criteria required a pre-implant LVEF of ≥50% and an anticipated ventricular pacing burden of over 20%. Exclusion criteria included a lack of pre-implant echocardiography, significant cardiac comorbidities (e.g., congenital heart disease, severe valve disorders, cardiomyopathy, or atrial fibrillation), or terminal illnesses with a life expectancy of less than one year.

Baseline characteristics, including electrocardiogram and echocardiographic data, were recorded for all patients. Clinical follow-up occurred every 3–6 months and involved monitoring for cardiac death or heart failure hospitalization. PICM was defined as a decline in LVEF by more than 10%, resulting in an LVEF of less than 50%, with RV pacing exceeding 20%, and no other identifiable cause, following established guidelines [3]. Composite outcomes included cardiac death (due to pump failure), HHF, PICM, and upgrades to biventricular pacing (BVP) during the follow-up period.

### 2.2 permanent pacemaker (PPM) Implantation

Patients were implanted with permanent pacemakers approved by the Korean Food & Drug Administration, using devices from Abbott (St. Paul, MN, USA) and Medtronic (Minneapolis, MN, USA). The implantation process followed standard procedures previously described [14]. Briefly, pacemaker leads were implanted via the axillary vein, with the RV leads positioned in the RV apex or mid-septum based on operator discretion. RV septum placement was facilitated using a Mond stylet (Abbott Laboratories, Chicago, IL, USA), fluoroscopic grid guidance, and biplane imaging.

Electrical parameters, including bipolar ventricular stimulation threshold, R wave amplitude, and lead impedance, were measured five minutes post-RV lead deployment. Acceptance criteria were an R wave >5 mV and a pacing threshold <1.5 V. Ventricular lead positions were confirmed using fluoroscopy in both left and right anterior oblique views. Baseline 12-lead electrocardiography (ECG) parameters were collected within 24 hours of implantation and regularly at follow-up visits (every six months). During pacemaker programming, efforts were made to reduce RV pacing burden by appropriately adjusting the atrioventricular (AV) delay.

### 2.3 Echocardiography

All patients underwent echocardiographic evaluations before implantation, within seven days post-implantation, and one year later. Images were obtained using a 2.5-MHz phased-array transducer (Vivid 9; GE Vingmed, Horton, Norway), following the American Society of Echocardiography guidelines. The LVEF was measured using the modified Simpson’s biplane method, while mitral inflow was assessed with pulsed wave Doppler ultrasonography. Diastolic function was evaluated using color tissue Doppler imaging, including the mitral annular septal E^′^ velocity, E/E^′^ ratio, tricuspid regurgitant jet velocity, and left atrial volume index (LAVI). Diastolic dysfunction was defined when more than half of these parameters met the cutoff values.

All echocardiographic measurements were performed using the same machine for baseline and follow-up exams. Two independent and experienced echocardiographers, blinded to prior results, assessed the images. GLS was measured using EchoPAC software (Version 113, GE Vingmed Ultrasound, Horten, Norway). Peak longitudinal strain was calculated automatically from the endocardial borders and tracked throughout the cardiac cycle.

### 2.4 Reproducibility of Data

Echocardiographic images underwent two separate analyses, spaced more than one month apart, conducted independently by two investigators, namely S.S. Kim and H.K. Jeong. Both analysts were kept unaware of the initial measurement results and each other’s findings. The intraclass correlation coefficients (ICC) values for all diameter measurements were >0.80, indicating good and excellent agreement. There was no statistically significant inter-observer variability for EF (ICCs = 0.833 (95% CI 0.696–0.908), *p* < 0.001) and GLS (ICCs = 0.857 (95% CI 0.763–0.914), *p* < 0.001) based on a significance level of >5%.

### 2.5 Statistical Analysis

Baseline characteristics were expressed as mean ± standard deviation for continuous variables and as frequencies with percentages for categorical variables. Group comparisons were conducted using Student’s *t*-test for continuous variables and the chi-squared test for categorical variables. Paired *t*-tests were used for within-group comparisons. Inter-observer agreement between the two echocardiographers was assessed using the ICC test with a 95% confidence interval (CI) in a two-way random model. Predictors of composite clinical outcomes were identified using a Cox proportional hazards regression model, incorporating variables with a univariate *p*-value < 0.2 to control for potential confounders. Receiver operating characteristic (ROC) curve analysis was used to determine cutoff values for the occurrence of composite outcomes. All statistical analyses were conducted using IBM SPSS Statistics (v. 26.0; IBM Corp., Armonk, NY, USA), with *p*-values < 0.05 considered statistically significant. Results are reported as hazard ratios (HR) with 95% CI.

## 3. Results

### 3.1 Study Population

**Fig. 1.**
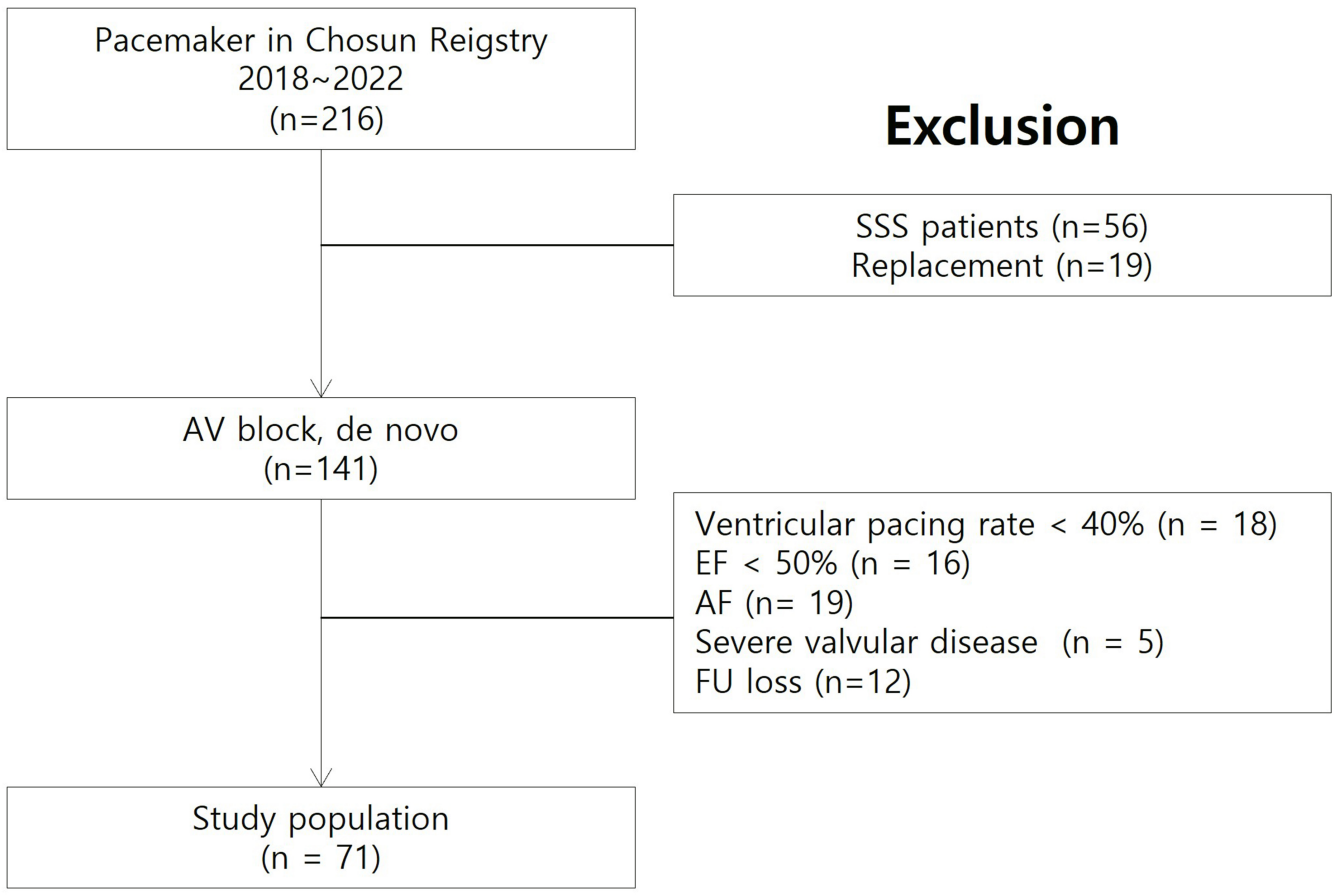
**Flow chart of the study protocol**. AV, atrioventricular; SSS, sick sinus syndrome; EF, ejection fraction; AF, atrial fibrillation; FU, follow up.

Between 2018 and 2022, 216 patients presenting with symptomatic bradycardia requiring permanent pacemaker implantation were screened for inclusion. The study selection process is illustrated in Fig. 1. After applying exclusion criteria, 71 patients (28 males, 39.4%; mean age 73 ± 9 years) were included in the final analysis. These patients were categorized into two groups based on the development of PICM. Table 1 summarizes the baseline clinical characteristics. No significant differences were observed between the groups in terms of age, gender, and cardiovascular risk factors, except for diabetes mellitus (18% vs. 57%, *p* = 0.041). The baseline LVEF was similar between the groups (62 ± 7 vs. 59 ± 8, *p* = 0.281); however, patients in the PICM group had significantly lower GLS (–19.7 ± 3.0 vs. –17.0 ± 3.4, *p* = 0.033) and longer paced QRS duration (158 ± 16 vs. 172 ± 10, *p* = 0.028).

**Table 1.** **Baseline characteristics**.

		Normal (N = 64)	PICM (N = 7)	*p* value
Age (years)		73.7 ± 9.3	67.1 ± 9.6	0.082
Male n (%)		24 (37.5%)	4 (57.1%)	0.422
Cardiovascular risk factor				
	Hypertension	43 (67.2%)	5 (71.4%)	0.820
	Diabetes mellitus	12 (18.8%)	4 (57.1%)	0.041 *
	Hyperlipidemia	19 (29.7%)	4 (57.1%)	0.203
	Coronary artery disease	7 (10.9%)	1 (14.3%)	0.584
	Cerebrovascular disorder	7 (10.9%)	0 (0 %)	0.467
	Chronic kidney disease	2 (3.1%)	1 (14.3%)	0.271
Echocardiography				
	Ejection fraction	62.2 ± 6.9	59.1 ± 8.2	0.281
Diastolic dysfunction				
	E/e’ >15	19 (30.2%)	3 (42.9%)	0.383
	e’ (septal) <7	46 (73.0%)	7 (100%)	0.183
	TR velocity >2.8 m/s	31 (48.4%)	0 (0%)	0.014 *
	LAVI >34 m^2^	37 (57.8%)	6 (85.7%)	0.153
	Global longitudinal strain	–19.7 ± 3.0	–17.0 ± 3.4	0.033 *
Electrocardiography				
	Paced QRS duration	157.9 ± 15.8	171.7. ± 10.2	0.028 *

LAVI, left atrial volume index; PICM, pacing-induced cardiomyopathy; TR, tricuspid regurgitation. *, *p* value <0.05.

### 3.2 Device-Related Parameters

Device characteristics for each group are presented in Table 2. No significant differences were found between the groups in terms of P and R wave amplitudes, pacing thresholds, or impedance values. None of the patients experienced peri-procedural complications such as stroke, myocardial infarction, or heart failure during pacemaker implantation. Additionally, no lead dislodgements, capture losses, infections, or embolisms occurred. The percentage of ventricular pacing was comparable between the groups.

**Table 2.** **Device related parameters**.

		Normal (N = 64)	PICM (N = 7)	*p* value
Atrial leads				
	Implant P wave	2.90 ± 1.25	3.18 ±1.03	0.577
	Pacing threshold	0.73 ± 0.24	0.67 ± 0.12	0.543
	Impedance	492.6 ± 119.1	469.5 ± 69.5	0.619
Ventricular leads				
	Implant R wave	10.46 ± 2.99	10.34 ± 2.48	0.915
	Pacing threshold	0.86 ± 0.42	0.57 ± 0.18	0.592
	Impedance	584.8 ± 98.3	596.4 ± 104.7	0.769
RV apical pacing		4 (6.3%)	1 (14.3%)	0.414
Ventricular pacing percentage (%)		96.7 ± 10.6	97.7 ± 3.20	0.849

PICM, pacing-induced cardiomyopathy; RV, right ventricular.

### 3.3 Serial LVEF and GLS Assessments

**Fig. 2.**
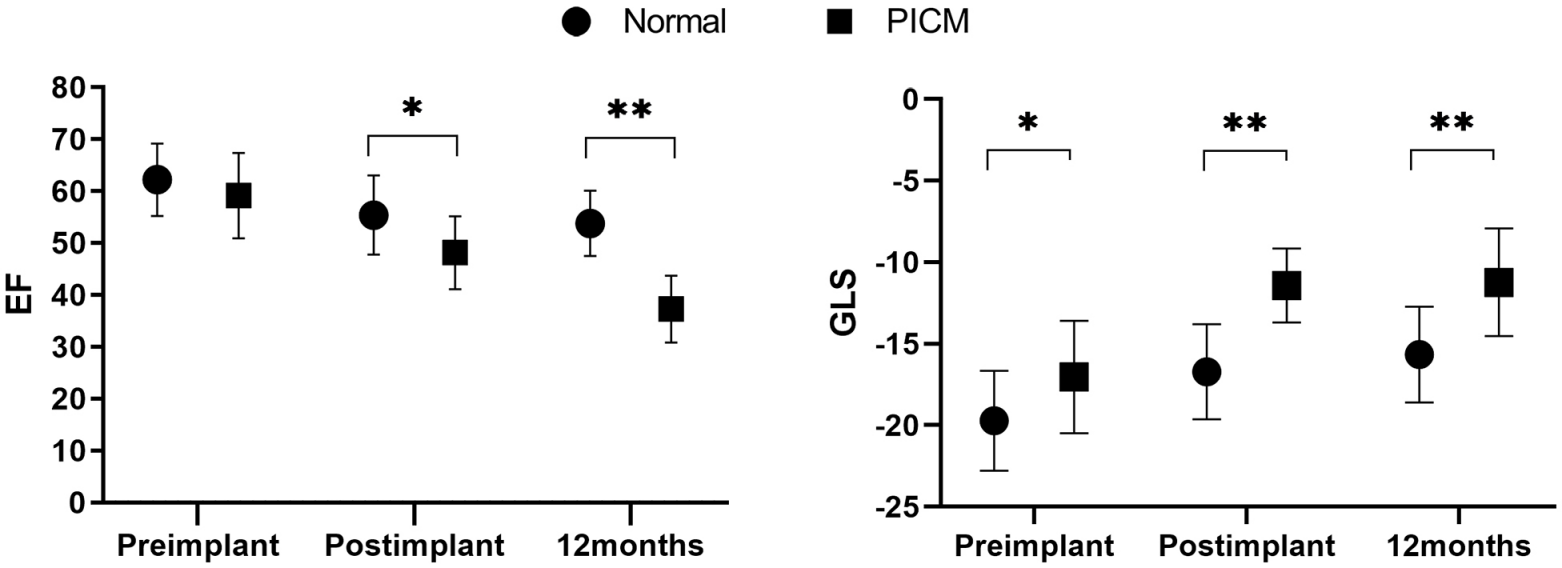
**Serial GLS and EF assessment**. Baseline EF were not different between the two groups (62 ± 6 vs. 59 ± 8, *p* = 0.373). However, post-implant EF were lower in the PICM group (55 ± 7 vs. 48 ± 7, *p* = 0.019). After 12 months, EF were significantly lower in the PICM group (53 ± 6 vs. 37 ± 6, *p* = 0.001). Baseline GLS were lower in the PICM group, unlike EF (–19.72 ± 3.06 vs. –17.04 ± 3.45, *p* = 0.033). Post-implant GLS, 12 months GLS were significantly lower in the PICM group (–16.72 ± 2.91 vs. –11.42 ± 2.27, *p* < 0.001) and (–15.66 ± 2.94 vs. –11.23 ± 3.3, *p* < 0.001), respectively. EF, ejection fraction; GLS, global longitudinal strain; PICM, pacing-induced cardiomyopathy. *, *p* value <0.05; **, *p* value <0.001.

Table 3 outlines the serial assessments of LVEF and GLS over time. Before pacemaker implantation, LVEF did not differ between the two groups (Fig. 2). However, post-implantation LVEF (within one week) was significantly lower in the PICM group (55 ± 7 vs. 48 ± 7, *p* = 0.019). At the 12-month follow-up, this difference persisted, with the PICM group showing a substantially reduced LVEF (53 ± 6 vs. 37 ± 6, *p* = 0.001). In terms of GLS, pre-implant values were lower in the PICM group compared to the non-PICM group (–19.7 ± 3.0 vs. –17.0 ± 3.4, *p* = 0.033). Post-implant GLS and 12-month GLS values were also significantly lower in the PICM group (–16.7 ± 2.9 vs. –11.4 ± 2.2, *p* < 0.001) and (–15.6 ± 2.9 vs. –11.23 ± 3.3, *p* < 0.001), respectively.

**Table 3.** **Serial assessment in LVEF and GLS**.

		Normal (N = 64)	PICM (N = 7)	*p* value
Ejection fraction (%)				
	Pre-implant	62.2 ± 6.99	59.14 ± 8.23	0.373
	Post-implant	55.38 ± 7.64	48.14 ± 7.03	0.019
	12 months	53.77 ± 6.30	37.29 ± 6.44	<0.001
Global longitudinal strain				
	Pre-implant	–19.72 ± 3.06	–17.04 ± 3.45	0.033
	Post-implant	–16.72 ± 2.91	–11.42 ± 2.27	<0.001
	12 months	–15.66 ± 2.94	–11.23 ± 3.3	<0.001

PICM, pacing-induced cardiomyopathy; LVEF, left ventricular ejection fraction; GLS, global longitudinal strain.

### 3.4 Composite Outcomes 

**Fig. 3.**
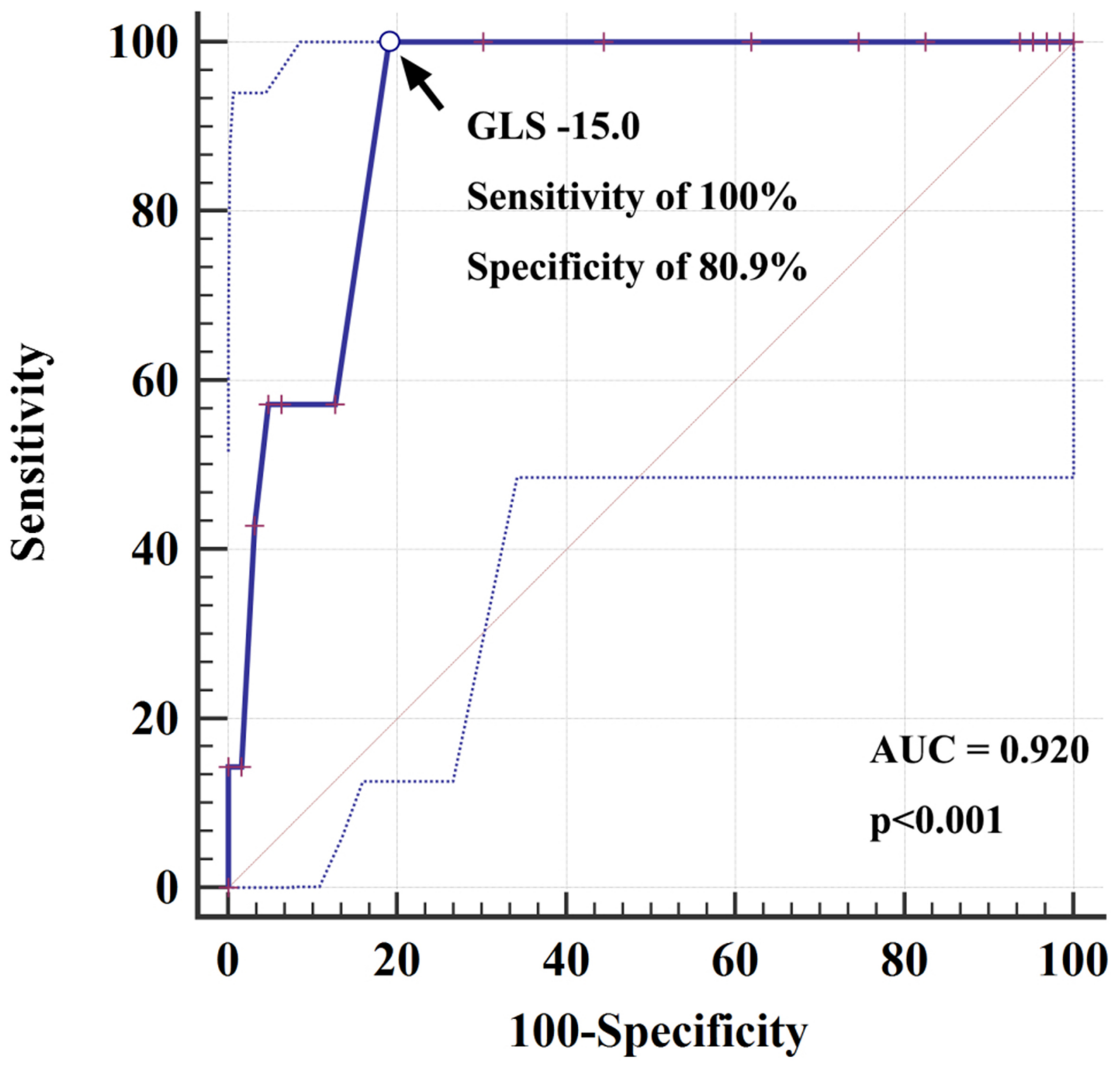
**Receiver operating characteristic (ROC) curve for global longitudinal strain (GLS) in predicting pacing-induced cardiomyopathy (PICM)**. The ROC curve analysis demonstrated an area under the curve (AUC) of 0.92 (95% CI 0.829–0.971; *p* < 0.001), indicating that a GLS value of –15.0 had a sensitivity of 100% and specificity of 80.9% for predicting the development of PICM.

The composite outcomes are summarized in Table 4. Over a median follow-up period of 3 years (1062 days, interquartile range 663–1392 days), 9.8% of the patients experienced combined outcomes, including 2 deaths, 6 hospitalizations, 7 PICM cases, and 3 upgrades to BVP. Table 5 presents the results of the Cox proportional regression analysis, identifying decreased post-implant GLS as an independent predictor of PICM (HR, 1.715; 95% CI, 1.174–2.504; *p* = 0.005). ROC curve analysis revealed an area under the curve (AUC) of 0.92 (95% CI 0.829–0.971, *p* < 0.001), indicating that a GLS value of –15.0 had 100% sensitivity and 80.9% specificity for predicting PICM (Fig. 3).

**Table 4.** **Composite outcomes**.

		Patient, n (%)
Follow up periods, days (IQR)		1062 (663–1392)
Composite clinical outcomes		7 (9.8 %)
	Cardiac death (Pump failure), n (%)	2 (2.8 %)
	Hospitalization due to HF, n (%)	6 (8.4%)
	PICM, n (%)	7 (9.8%)
	BVP upgrade, n (%)	3 (4.2%)

BVP, biventricular pacing; HF, heart failure; PICM, pacing-induced cardiomyopathy; IQR, interquartile range.

**Table 5.** **Predictors of composite clinical outcomes using the Cox proportional hazard model**.

	Univariate analysis			Multivariate analysis		
	HR	95% CI	*p* value	HR	95% CI	*p* value
Age	0.918	0.842–1.002	0.056			
Gender (Male)	0.295	0.054–1.612	0.159			
Diabetic mellitus	4.272	0.926–19.707	0.063			
Hyperlipidemia	3.073	0.673–14.038	0.148			
Chronic kidney disease	9.787	1.132–84.646	0.038			
Post-implant EF	0.917	0.838–1.005	0.064			
Pre-implant GLS	1.253	0.963–1.631	0.093			
Post-implant GLS	1.683	1.236–2.287	0.001	1.715	1.174–2.504	0.005
Paced QRS duration	1.111	1.020–1.210	0.016			

GLS, global longitudinal strain; HR, hazard ratio; CI, confidence interval; EF, ejection fraction.

## 4. Discussion

Chronic RV pacing can lead to significant deterioration in left ventricular function due to artificial electromechanical dyssynchrony [1, 2]. In some cases, patients may experience left ventricular dysfunction after PPM implantation without a clear cause, a condition referred to as PICM. The reported incidence of PICM varies between 10–20% over 3–4 years post-PPM implantation [15, 16, 17]. This variability is most likely due to differences in PICM definitions, patient population characteristics, and follow-up duration. In our study, we observed an overall PICM incidence of 9.8% over a median follow-up period of three years, using current definitions [3].

PICM has been associated with an increased risk of AF, heart failure hospitalizations, and cardiac mortality [18]. Although the exact mechanisms underlying PICM are not fully understood, ventricular dyssynchrony is thought to play a central role. RV pacing disrupts the normal timing of ventricular contraction, with early-activated regions of the LV contracting prematurely while late-activated segments contract abnormally late. This dyssynchrony leads to mechanical inefficiency and strain redistribution, which over time, may result in reduced LVEF and heart failure [19, 20]. Prolonged RV pacing exacerbates this effect, contributing to the development of cardiomyopathy through electrical and mechanical dyssynchrony.

Several factors are known to predict the development of PICM, including RV apical pacing, increased pacing burden, lower baseline LVEF, and prolonged QRS duration. Other predictors include older age [21], male gender [22], AF [15], diastolic dysfunction [23], and abnormal GLS [13]. Notably, reduced EF is a critical risk factor for PICM, as demonstrated in studies such as the DAVID trial [2], where patients with a high RV pacing burden (>40%) had significantly higher rates of death or HF hospitalization. Current guidelines recommend regular echocardiographic monitoring for patients with reduced EF undergoing PPM, but there is limited guidance for patients with preserved EF, such as those included in our study.

Our study contributes to the literature by focusing on the early changes in GLS in patients with preserved EF following pacemaker implantation. We observed a noticeable reduction in both GLS and LVEF in patients who developed PICM, even in those with initially preserved EF. GLS, as a marker of subclinical LV dysfunction, showed reductions before significant declines in LVEF, underscoring its sensitivity as an early indicator of PICM. These findings suggest that routine GLS assessment could facilitate earlier detection of PICM, guiding timely clinical intervention and more frequent echocardiographic surveillance. A previous study [13] has highlighted the association between GLS and PICM, but our study emphasizes the importance of monitoring early post-implantation changes, particularly in patients with preserved EF.

In addition, our study reinforces the role of GLS as a predictive marker in clinical practice. A post-implant GLS value of <–15.0 demonstrated high sensitivity (100%) and specificity (80.9%) in predicting the development of PICM. This reinforces the need for integrating GLS monitoring into routine follow-up protocols, especially for patients at heightened risk of PICM. Early identification of abnormal GLS values should prompt closer follow-up and consideration of guideline-directed medical therapy or BVP upgrades where appropriate.

Interestingly, we observed that LVEF continued to decline over the 12-month follow-up period, while GLS remained relatively stable after an initial reduction in the PICM group. This finding may be explained by the different characteristics of GLS and LVEF as markers of LV dysfunction. GLS, being a more sensitive marker, detects early subclinical changes, whereas LVEF reflects global systolic function, which declines more gradually. This temporal discrepancy suggests that GLS is an earlier and more sensitive indicator of PICM compared to LVEF. Consequently, incorporating both GLS and LVEF into routine monitoring protocols can provide a more comprehensive assessment of LV function at different stages of disease progression.

**Fig. 4.**
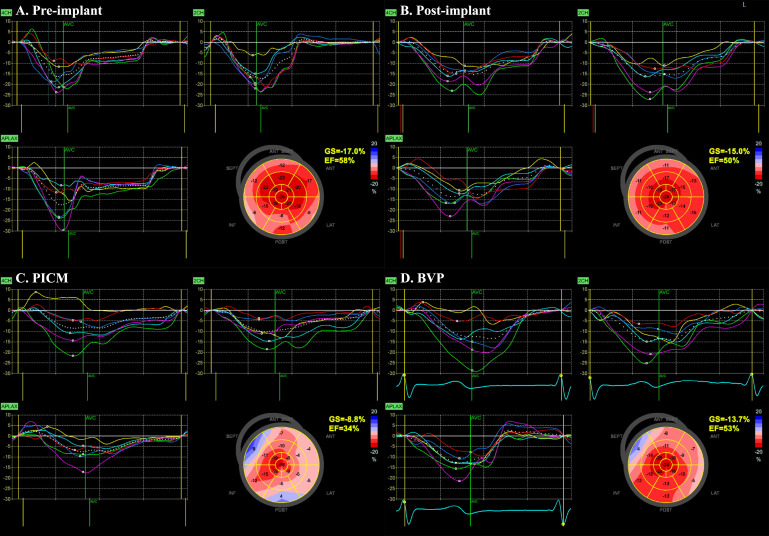
**Representatives of GLS pattern in patients with PICM**. (A) Pre-implant GLS of –17.0%, showing a normal pattern with nearly simultaneous inward contraction of the septum and lateral wall. (B) Post-implant GLS of –15.0%, illustrating a double-peak septal strain pattern. The septum contracts early, causing pre-stretching of the lateral wall, followed by lateral wall contraction and septal rebound stretch. (C) In a patient who developed PICM, GLS dropped to –8.8%. A heterogeneous pattern is observed with no septal rebound stretch or septal flash. The EF decreased to 34%. (D) Following biventricular pacing upgrade, GLS improved, showing a nearly simultaneous inward contraction of the septum and lateral wall. EF also improved, reaching 53%. EF, ejection fraction; GLS, global longitudinal strain; PICM, pacing-induced cardiomyopathy; AVC, aortic valve close; BVP, biventricular pacemaker; ANT, anterior; SEPT, septum; LAT, lateral; POST, posterior; INF, inferior; CH, chamber; APLAX, apical long axis; GS, global longitudinal strain.

Reversing PICM may be achievable by improving ventricular dyssynchrony. In addition to guideline directed medical therapy (GDMT) for heart failure with reduced EF, alternative pacing strategies have been proposed. These include upgrading to BVP or more physiological pacing techniques such as His bundle pacing or left bundle branch pacing, which are known to minimize ventricular dyssynchrony. Several studies have shown the efficacy of BVP in improving HF symptoms and promoting reverse remodeling of the left ventricle in patients with PICM [24, 25, 26]. Current guidelines recommend upgrading to BVP in patients with significant ventricular pacing burden who experience a decline in LV function or worsening HF symptoms [3, 24, 25, 26, 27, 28]. In our study, three patients underwent BVP upgrades, all of whom exhibited improved LVEF and normalization of the heterogeneous strain pattern after the upgrade (Fig. 4).

In conclusion, our findings highlight the importance of early GLS monitoring in patients with preserved EF undergoing pacemaker implantation. Early detection of reductions in GLS may allow for timely clinical intervention and potentially improve outcomes through GDMT or pacing upgrades. Further studies, particularly larger multicenter trials, are needed to validate these findings and explore long-term outcomes associated with GLS-guided management strategies.

### Limitations

Our study has several limitations that should be acknowledged. First, this investigation was conducted at a single medical center in South Korea, with a relatively small sample size of 71 patients, of whom only 7 reached the primary outcome. This small sample size may limit the generalizability of our findings to broader populations. While the results showed statistical significance, the small cohort size restricts the overall applicability and robustness of the conclusions, particularly regarding the relationship between PICM and GLS. Larger, multicenter studies are needed to validate these findings and further explore the association across more diverse patient populations.

Additionally, the nonrandomized nature of this registry introduces the potential for selection bias. This limitation is exacerbated by the exclusion of patients who were hospitalized or died from causes unrelated to heart failure (e.g., sepsis, cancer, and cerebrovascular accidents). The exclusion of these patients may have influenced the primary outcome and could affect the comprehensive understanding of GLS’s predictive power in various clinical scenarios. Furthermore, patients who initially received temporary pacemakers in the emergency room, as well as those without pre-implant echocardiography due to the use of portable echocardiography, were excluded. This could have further limited the study’s comprehensiveness and applicability to real-world settings where such conditions are common.

Another important limitation is that this study did not include patients who underwent conduction system pacing, such as His bundle pacing or left bundle branch area pacing, which have been shown to improve LVEF and heart failure symptoms in patients with PICM. At the time of our study, these techniques were not yet widely adopted in South Korea, but their exclusion limits our ability to fully assess newer pacing strategies that may reduce the incidence of PICM.

Despite these limitations, it is important to note that there is a lack of published data on GLS and its role in predicting PICM, making our study a valuable preliminary contribution to this area of clinical research. However, a cautious interpretation of our results is necessary, given the inherent limitations. Future research involving larger, multicenter trials and diverse patient populations will be crucial in validating our findings and determining the best approaches for identifying and managing individuals at risk for PICM, particularly those with reduced GLS.

## 5. Conclusions

PICM is a notable complication associated with permanent RV pacing, even in patients with preserved LVEF. Over our three-year observation period, RV pacing significantly increased the risk of PICM in these patients. Importantly, post-implant GLS was found to be a more reliable indicator of PICM risk than pre-implant GLS, with a post-implant GLS threshold of <–15 identified as a critical predictor. Patients exhibiting reductions in GLS after pacemaker implantation are at elevated risk for developing PICM and should undergo more frequent echocardiographic monitoring.

Regular follow-up and early intervention are essential for these high-risk patients. When GLS falls below the threshold of –15, clinical interventions, such as early initiation of GDMT and consideration of BVP upgrades, should be explored. The timely implementation of these strategies may prevent further deterioration and improve patient outcomes.

Future research should aim to validate these findings in larger, multicenter studies across diverse populations. Additionally, long-term follow-up is needed to assess the outcomes of GLS-guided interventions, which could further refine the management of patients at risk for PICM.

## Availability of Data and Materials

The datasets used and/or analyzed during the current study are available from the corresponding author on reasonable request.
